# Cancer Genomic Alterations Can Be Potential Biomarkers Predicting Microvascular Invasion and Early Recurrence of Hepatocellular Carcinoma

**DOI:** 10.3389/fonc.2022.783109

**Published:** 2022-01-27

**Authors:** Zhaodan Xin, Jin Li, Haili Zhang, Yi Zhou, Jiajia Song, Piaopiao Chen, Ling Bai, Hao Chen, Juan Zhou, Jie Chen, Binwu Ying

**Affiliations:** ^1^ Department of Laboratory Medicine, West China Hospital, Sichuan University, Chengdu, China; ^2^ Med+ Molecular Diagnostics Institute of West China Hospital/West China School of Medicine, Chengdu, China; ^3^ West China School of Medicine, Sichuan University, Chengdu, China; ^4^ Department of Liver Surgery & Liver Transplantation Center, West China Hospital, Sichuan University, Chengdu, China

**Keywords:** hepatocellular carcinoma, microvascular invasion, early recurrence, cancer genome mutations, circulating tumor DNA

## Abstract

**Background:**

High recurrence incidence and poor survival after hepatectomy are enormous threats to hepatocellular carcinoma (HCC) patients, which can be caused by microvascular invasion (MVI). However, it is difficult to predict preoperative MVI status. In this study, we focus on cancer genomic alterations to comprehensively explore potential MVI and early recurrence biomarkers and provide clues to the mechanisms of HCC invasion and metastasis.

**Methods:**

Forty-one patients with initially suspected HCC who were undergoing hepatectomy were finally enrolled. High-throughput targeted sequencing was performed on genomic alterations in their preoperative plasma and surgical fresh tumor tissues utilizing the 1,021-gene panel.

**Results:**

HCC patients without MVI had longer RFS than MVI ones (*p* < 0.0001). The mutant incidence of genes like *KEAP1, TP53*, *HIST1H3D*, *NFKBIA*, *PIK3CB*, and *WRN* was higher in both MVI and early-recurrence patients than their counterparts. Besides, the alteration rates of Rap1 and Ras signaling pathways were significantly higher in MVI patients than NMVI ones (*p* < 0.05), and a similar trend of differences was also found in early-recurrence/non-recurrence comparison. The maximal variant allele frequency (VAF) of circulating tumor DNA (ctDNA) was statistically higher in MVI patients than NMVI ones (0.038 vs. 0.012, *p* = 0.0048). With the cutoff value of 0.018, ctDNA maximal VAF could potentially predict the presence of MVI with an AUC of 0.85 (95% CI 0.693–0.998, *p* = 0.0062).

**Conclusion:**

The integration of a panel containing specific mutated genes and ctDNA maximal VAF for predicting MVI and early recurrence of HCC may achieve better performance.

## Introduction

Hepatocellular carcinoma (HCC), accounting for the majority of primary liver cancer, is one of the most common malignancies and the third leading cause of cancer death worldwide (1). Despite the diverse development of multiple therapeutic regimens, surgical liver resection is the preferred choice for patients with solitary tumors at early stages (2, 3). Unfortunately, high recurrence incidence after curative treatment is still a major challenge for HCC patients (4–6).

The enormous hindrance to improve prognosis may derive from the fact that HCC cells can penetrate the microvasculature and are prone to evolve vascular invasion and intrahepatic metastasis. Microvascular invasion (MVI) has increasingly been recognized to be one of the most significant risk factors for HCC recurrence and metastasis after hepatectomy or liver transplantation (7–9). Moreover, many scholars have devoted themselves to exploring the optimal surgical strategy selection (10–12) according to MVI status, while currently, the identification of MVI is still based on histopathological examination after surgical resection. Therefore, preoperative prediction of MVI presence is urgent.

Moreover, as the tumor recurrence in patients with HCC after hepatic resection is a major cause of post-resection death (13), it is vital to identify HCC patients at high risk of recurrence to strengthen surveillance. Researchers have investigated many methods and biomarkers to predict early recurrence of HCC patients after surgery, while the efficacy was limited and the cutoff time was usually set as the second year after resection. However, some researchers have found that recurrence within the first year after resection was particularly aggressive to expect a poor prognosis (4, 14). As early recurrence of HCC is considered closely correlated with MVI status, more reliable results might be obtained *via* the comprehensive analysis of MVI and early recurrence predictive biomarkers at the same time.

Recently, studies have made attempts on the preoperative prediction of MVI or prognosis in HCC such as genomic mutation biomarkers that shed light on the molecular mechanisms underlying HCC progression and prognosis (15, 16). In view of these research results, some gene mutations can be potential predictors of HCC prognosis, playing an important role in HCC carcinogenesis and metastasis. Furthermore, the function of mutated genes as regulators of signaling pathways deserves further exploration to provide clues for prognosis. Herein, we decide to investigate more extensively on mutated genes and signaling pathways in addition to what has been reported, and then preliminarily exploit possible mechanisms facilitating tumor invasion and metastasis. In addition, circulating tumor DNA (ctDNA) can reflect tumor tissue genomic mutations, providing information for diagnosis or prognostic prediction (17, 18). Besides, ctDNA can be obtained in a noninvasive way before hepatectomy, providing potential assistance in preoperative decision-making. Hence, we will also explore whether preoperative plasma ctDNA of HCC patients could become MVI predictive biomarkers or not.

## Materials and Methods

### Ethics Statement

All patients in the study provided written informed consent, and the study was approved by Biomedical Ethics Committee of West China Hospital of Sichuan University [Reference No. 2019 (203)].

### Subjects

A total of 44 patients with initially suspected HCC who were undergoing hepatectomy at West China Hospital of Sichuan University in 2019 were prospectively recruited. Surgical details of these patients are listed in **Supplementary Table 3**.

The inclusion and exclusion criteria of patients were as follows (1) definitive pathological diagnosis of HCC without other tumors; (2) no macrovascular invasion was found; (3) no TACE (Transcatheter Arterial Chemoembolization) or radiofrequency treatment and other anti-tumor treatment before; and (4) TNM Stage I or II HCC. After postoperatively pathological diagnosis, all samples were enrolled in the high-throughput sequencing experiment. Patients with pathologically proven microvascular recidivism, vascular tumor thrombus, and tumor thrombus and without macrovascular invasion were regarded as MVI. Those who relapse within 1 year (≤12 months) after surgery were regarded as early recurrence. After the exclusion of 1 patient who was lost to follow-up and 2 patients with failed sequencing, 41 HCC patients were enrolled eventually (**Supplementary Table 1**).

### Peripheral Blood and Tumor Tissues Collection

Twenty milliliters of fresh EDTA anticoagulated peripheral blood samples of patients was collected before hepatectomy, and the plasma was separated within 2 h and then stored at −80°C. The plasma separation process includes (a) centrifugation at 1,600 *g* for 10 min at 4°C and aspirate upper layer of plasma, and the middle blood leukocyte layer was aspirated to a corresponding new cryotube, and (b) centrifugation of aspirated plasma at 16,000 *g* for 10 min at 4°C, and the supernatant was transferred to cryotubes as the plasma sample. Fresh tumor tissues that were not less than 60 mg were resected during the surgery. The MVI diagnosis of each sample was independently determined by two senior pathologists.

### DNA Extraction

DNA from fresh tumor tissues was extracted using the QIAamp DNA Mini kit (QIAGEN, Germany) according to the manufacturer’s instructions. Correspondingly, circulating free DNA (cfDNA) from plasma was extracted utilizing the QIAamp Circulating Nucleic Acid kit (QIAGEN, Germany) following the manufacturer’s protocol. Specifically, peripheral blood leukocyte (PBL) DNA was extracted for germline mutation filtering by using QIAamp DNA Mini Blood kit (QIAGEN, Germany). Qubit 3.0 (Qubit, USA) was utilized to preliminarily quantify the extracted DNA, then agarose electrophoresis was used to determine the degree of DNA degradation and contamination. Besides, Agilent 2100 Bioanalyzer (Agilent, USA) was used to obtain the size of cfDNA fragments.

### DNA Library Construction, Targeted DNA Sequencing, and Bioinformatic Data Analysis

DNA purified with 1.5× magnetic beads after fragmentation was prepared from tumor tissues and PBLs for library construction. Germline, tumor tissue, and ctDNA libraries were prepared utilizing the human 1,021 gene mutation detection kit (Gene+, China). Capture probe was programmed to span the coding sequences or exon hotspots of the 1,021 genes frequently mutated in solid tumors, covering all 4,847 exons of 312 genes, as well as introns, promoters, and fusion breakpoint regions of 38 genes and 1,778 coding regions of 709 other related genes (**Supplementary Table 2**). Libraries were hybridized to these custom-designed biotinylated oligonucleotide probes. Sequencing was performed with the Gene+Seq-2000 sequencer (Gene+, China) according to the manufacturer’s instructions, with the sequencing depth over 500×, 2000×, and 300×, respectively. After removing terminal adaptor sequences and low-quality reads (>10% N rate, >50% bases with Q ≤ 5), the remaining clean reads were mapped to the reference human genome (hg19) through the Burrows-Wheel Aligner (BWA) software with default parameters, followed by removing duplicate reads using Gene Analysis Toolkit (GATK, https://www.broadinstitute.org/gatk/). Single-nucleotide variants (SNVs) and somatic insertions/deletions (InDel) were called utilizing MuTect2 algorithm (https://software.broadinstitute.org/gatk/documentation/tooldocs/3.8-0/org_broadinstitute_gatk_tools_walkers_cancer_m2_MuTect2.php), and the gnomAD database was applied to filter germline mutations. After mutation calling, mutated variant allele frequency (VAF) ≥0.01 was filtered.

### Follow-Up and Tumor Recurrence

All patients were followed up regularly for more than 1 year after surgery. They were scheduled with liver function tests, tumor marker tests, and imaging examinations [including computed tomography (CT) scan, magnetic resonance imaging (MRI) scan, or abdominal ultrasonic examination]. Recurrence was diagnosed by imaging examinations. The time to recurrence was defined as the interval between hepatectomy and the diagnosis of intrahepatic recurrence or extrahepatic metastasis. Follow-up was terminated on May 23, 2021. The mean follow-up time was 15.86 ± 6.62 months (median, 18.70 months; range, 1.37–23.27 months). Recurrence within 1 year after surgery were defined as an early recurrence (19).

### Statistical Analysis

Continuous variables are expressed as the mean ± SD (standard deviation) and were compared using Student’s ***t***-test; categorical variables are expressed as frequencies (percentages) and were compared using *χ* ^2^ or Fisher’s exact test. The maftools package of R was exploited to perform a panoramic display of tumor tissue and ctDNA mutations in all samples. The association between MVI status and recurrence-free survival (RFS) was calculated using the Kaplan–Meier method and compared using the log-rank test. *χ* ^2^ or Fisher’s exact test was performed in MVI/NMVI groups and recurrence/non-recurrence groups to find the differentially mutated genes, and mutated genes in the MVI or recurrence group were annotated using the STRING database (version 11.0) (https://string-db.org/) for functional Gene Ontology (GO) and Kyoto Encyclopedia of Genes and Genomes (KEGG) pathway enrichment analysis. Several signaling pathways related to invasion and metastasis with false discovery rate (FDR) <0.05 were selected to analyze whether they were associated with MVI or early recurrence. If one or more genes mutated in the signaling pathway, the pathway was designated as mutated. VAF was defined as the ratio of the number of reads that supported the mutation to the total number of reads at the mutation site. We used the maximal VAF in ctDNA to reflect mutation load, and Mann–Whitney *U*-test was used to compare the difference between groups. Receiver operating characteristic curve (ROC) analysis was conducted to assess the performance of ctDNA VAF in the preoperative prediction of MVI. In all analyses, *p*-value of <0.05 was defined as statistically significant. All analyses were conducted using IBM SPSS (version 26.0, for Mac), GraphPad Prism (version 9.0.2, for Mac) software, and R software (version 4.0.5, for Mac).

## Results

### Clinicopathological Characteristics of HCC Patients

The detailed clinicopathological characteristics of our HCC cohort are demonstrated in **Table 1**. In HCC patients, the average age at diagnosis was 56.71 years old while most of them were male individuals (82.93%). In the preoperative serum examination, 32 patients (78.05%) displayed positive HBV surface antigen (HBs‐Ag) and ≥1.00E+02 IU/ml HBV-DNA was detected in 22 (53.66%) of the cohort. The mean tumor diameter was 5.62 ± 3.74 cm (range, 1.3–16.2 cm). Patients were classified according to Barcelona Clinic Liver Cancer (BCLC) stage. Thirty-five (85.37%) of them were classified as 0 or A while 6 (14.63%) patients were classified as B. Besides, 13 patients (31.71%) were determined MVI positive. Among them, 9 patients had MVI ≤5 and 4 patients had MVI whose number was more than 5. In the imaging results, 13 patients (31.71%) were found to have recurrence with imaging metastasis characteristics including intrahepatic and extrahepatic within 12 months.

**Table 1:** Clinicopathological characteristics of the HCC cohort.

Clinicopathological Variables	HCC patients (*N* = 41)	MVI group (*N* = 13)	NMVI group (*N* = 28)	*p*-value	Recurrence group (*N* = 13)	Non-recurrence group (*N* = 28)	*p*-value
Gender							
Male *n* (%)	34 (82.93%)	12 (92.31%)	22 (78.57%)	0.521	12 (92.31%)	22 (78.57%)	0.521
Female *n* (%)	7 (17.07%)	1 (7.69%)	6 (21.43%)		1 (7.69%)	6 (21.43%)	
Age (years) mean ± SD	56.71 ± 11.33	56.54 ± 12.17	56.79 ± 11.15	0.949	54.38 ± 11.66	57.79 ± 11.22	0.378
Height (cm) mean ± SD	164.98 ± 7.31	164.69 ± 8.49	165.11 ± 6.87	0.868	165.31 ± 4.63	164.82 ± 8.34	0.812
Weight (kg) mean ± SD	65.96 ± 12.14	65.15 ± 9.75	66.34 ± 13.25	0.775	66.85 ± 8.96	65.55 ± 13.49	0.755
ALT (IU/L) mean ± SD	53.10 ± 69.16	51.08 ± 49.95	54.04 ± 77.29	0.900	53.62 ± 53.15	52.86 ± 76.36	0.974
AST (IU/L) mean ± SD	57.27 ± 89.45	70.08 ± 94.23	51.32 ± 88.28	0.539	64.92 ± 94.42	53.71 ± 88.61	0.714
Total bilirubin (μmol/L) mean ± SD	13.79 ± 6.69	12.03 ± 3.51	14.61 ± 7.66	0.148	14.64 ± 6.30	13.40 ± 6.95	0.587
Direct bilirubin (μmol/L) mean ± SD	5.07 ± 2.99	4.62 ± 1.97	5.28 ± 3.37	0.516	5.45 ± 2.78	4.89 ± 3.11	0.580
Indirect bilirubin (μmol/L) mean ± SD	8.72 ± 4.00	7.42 ± 2.23	9.33 ± 4.51	0.157	9.19 ± 3.99	8.51 ± 4.07	0.620
HBV‐DNA (IU/ml)							
≥1.00E+02 *n* (%)	22 (53.66%)	9 (69.23%)	13 (46.43%)	0.173	9 (69.23%)	13 (46.43%)	0.173
<1.00E+02 *n* (%)	19 (46.34%)	4 (30.77%)	15 (53.57%)		4 (30.77%)	15 (53.57%)	
HBs‐Ag							
Positive *n* (%)	32 (78.05%)	9 (69.23%)	23 (82.14%)	0.600	9 (69.23%)	23 (82.14%)	0.600
Negative *n* (%)	9 (21.95%)	4 (30.77%)	5 (17.86%)		4 (30.77%)	5 (17.86%)	
AFP (ng/ml) mean ± SD	152.99 ± 298.15	287.10 ± 435.98	133.05 ± 280.92	0.18	104.56 ± 154.13	444.85 ± 1,224.34	0.196
PIVKA-II (mAU/ml) mean ± SD	4,677.00 ± 13,541.05	13,230.46 ± 22,187.92	705.75 ± 1,123.40	0.065	5,759.15 ± 11,818.93	4,174.57 ± 14,447.69	0.732
CEA (ng/ml) mean ± SD	2.60 ± 2.38	2.95 ± 1.35	2.44 ± 2.72	0.337	2.37 ± 1.17	2.71 ± 2.81	0.680
**Pathological results**							
Satellite focus							
Yes *n* (%)	2 (4.88%)	2 (15.38%)	0 (0%)	0.095	1 (7.69%)	1 (3.57%)	0.539
No *n* (%)	39 (95.12%)	11 (84.62%)	28 (100%)		12(92.31%)	27 (96.43%)	
Tumor diameter (cm) mean ± SD	5.62 ± 3.74	8.97 ± 4.59	4.07 ± 1.86	0.002*	7.52 ± 4.57	4.74 ± 2.98	0.025*
Tumor number							
Single *n* (%)	38 (92.68%)	11 (84.62%)	27 (96.43%)	0.232	11 (84.62%)	27 (96.43%)	0.232
Multiple *n* (%)	3 (7.32%)	2 (15.38%)	1 (3.57%)		2 (15.38%)	1 (3.57%)	
Differentiation							
Poor differentiation *n* (%)	14 (34.15%)	6 (46.15%)	8 (28.57%)	0.453	8 (61.54%)	6 (21.43%)	0.030*
Moderate differentiation *n* (%)	27 (65.85%)	7 (53.85%)	20 (71.43%)		5 (38.46%)	22 (78.57%)	
Cirrhosis							
Yes *n* (%)	15 (36.59%)	5 (38.46%)	10 (35.71%)	1	5 (38.46%)	10 (35.71%)	1
No *n* (%)	26 (63.41%)	8 (61.54%)	18 (64.29%)		8 (61.54%)	18 (64.29%)	
MVI							
Yes *n* (%)	13 (31.71%)	/		/	8 (61.54%)	5 (17.86%)	0.015*
No *n* (%)	28 (68.29%)				5 (38.46%)	23 (82.14%)	
MVI number							
>0 and ≤5 *n* (%)	9 (21.95%)	/		/	4 (30.77%) 4 (30.77%) 5 (38.46%)	5 (17.86%) 0 (0%) 23 (82.14%)	0.002*
>5 *n* (%)	4 (9.76%)					
0	28 (68.29%)					
**Imaging results**							
Cirrhosis							
Yes *n* (%)	17 (41.46%)	6 (46.15%)	11 (39.29%)	0.678	6 (46.15%)	11 (39.29%)	0.678
No *n* (%)	24 (58.54%)	7 (53.85%)	17 (60.71%)		7 (53.85%)	17 (60.71%)
Portal hypertension							
Yes *n* (%)	18 (43.90%)	7 (53.85%)	11 (39.29%)	0.382	7 (53.85%)	11 (39.29%)	0.382
No *n* (%)	23 (56.10%)	6 (46.15%)	17 (60.71%)		6 (46.15%)	17 (60.71%)
BCLC staging							
0/A *n* (%)	35 (85.37%)	9 (69.23%)	26 (92.86%)	0.129	10 (76.92%)	25 (89.29%)	0.57
B *n* (%)	6 (14.63%)	4 (30.77%)	2 (7.14%)		3 (23.08%)	3 (10.71%)
Recurrence (≤12 months)							
Yes *n* (%)	13 (31.71%)	8 (61.54%)	5 (17.86%)	0.015*	/		/
No *n* (%)	28 (68.29%)	5 (38.46%)	23 (82.14%)

* and bold font indicate p-value ＜ 0.05.

### The Correlation of MVI and Recurrence

**Figure 1:**
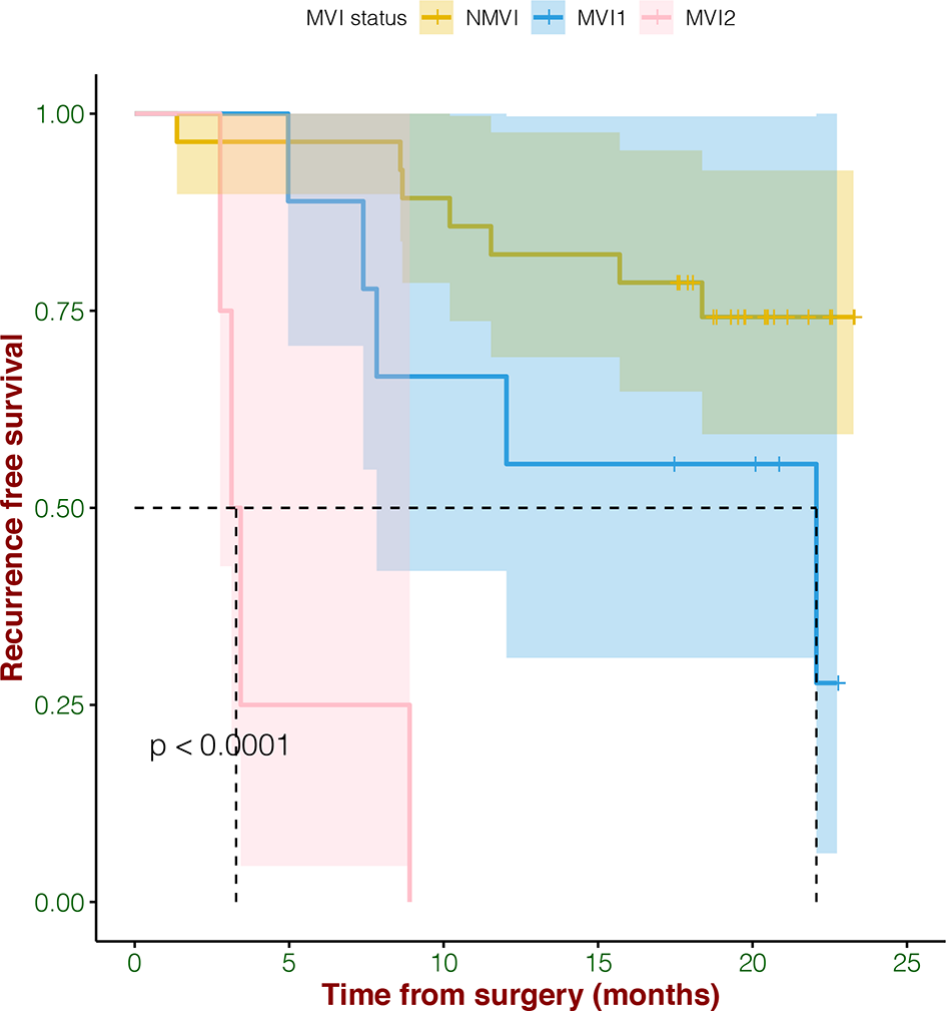
The correlation of MVI status and recurrence-free survival.

In survival analysis, MVI status including MVI1 (the number is >0 and ≤5), MVI2 (the number is >5), and NMVI demonstrated significant correlation with RFS (*p* < 0.0001) (**Figure 1**). What we found was that HCC patients without MVI had longer RFS, while with the severity of MVI increasing, the RFS was poorer.

### Tumor Tissues and ctDNA Genomic Landscape of HCC Patients

**Figure 2:**
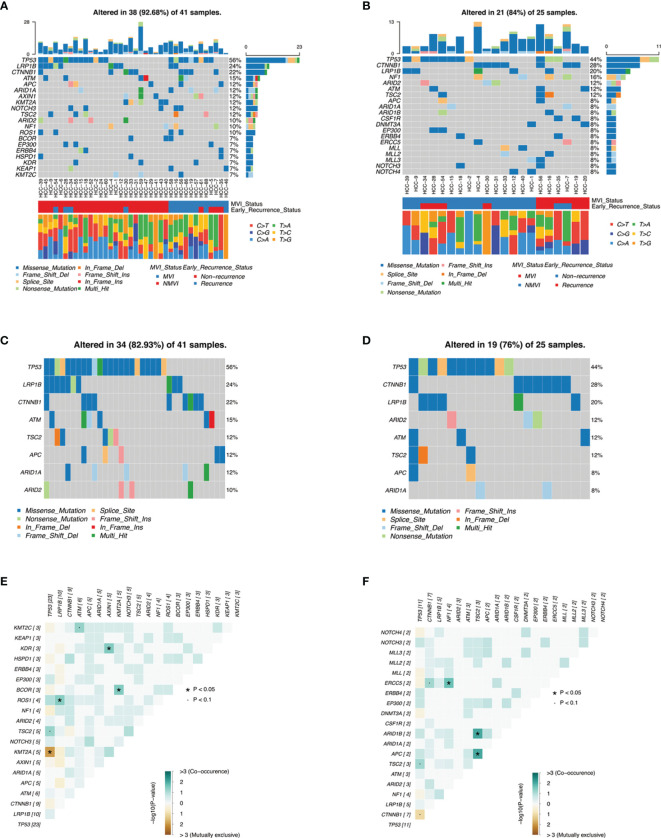
Genomic landscape of mutations in HCC cohorts. **(A, B)** are heatmaps illustrating top 20 mutant genes of tumor tissues and ctDNA samples in our HCC patients, respectively, with MVI status groups and early-recurrence status groups as well as guanine transformation types. **(C, D)** are heatmaps illustrating concordant genes between top 20 mutant genes of tumor tissues and top 20 mutant genes of ctDNA, respectively. **(E, F)** demonstrate somatic interactions *via* exclusive/co-occurrence event analysis on top 20 mutated genes in tumor tissue samples and ctDNA samples, respectively.

Gene mutations in tumor tissues and plasma ctDNA were all analyzed. Mutations were detected in all tissue samples (41/41, 100%), in which a total of 181 genes were found mutated, involving 311 mutation sites. The top 20 genes with the highest mutation rates were illustrated, covering 92.68% of HCC patients (**Figure 2A**), including genes such as *TP53* (56%), *LRP1B* (24%)*, CTNNB1* (22%), *ATM* (15%), *APC* (12%), *ARID1A* (12%), *AXIN1* (12%), *NOTCH3* (12%), *TSC2* (12%), and *ARID2* (10%). The results of top mutant genes were similar to that queried in the COSMIC database (**Supplementary Figure 2A**).

Meanwhile, a total of 25 cases (25/32, 78.13%) of HCC patients were detected with ctDNA mutations. The top 20 genes with the highest mutation rates were also demonstrated (**Figure 2B**), and the most frequent mutations of ctDNA were similar to tumor tissues such as *TP53* (44%), *CTNNB1* (28%), and *LRP1B* (20%). These concordant mutated genes altered in 82.93% samples of tumor tissues and in 76% samples of plasma ctDNA, respectively, resulting in moderate consistency (**Figures 2C, D**).

Among the 311 genomic alterations of 181 genes from tumor tissues, most of them were SNP with missense mutation. Among 295 SNV mutations of base pair substitutions, T>A conversion (27.80%, 82/295) was the most frequent mutation, followed by C>T conversion (24.41%, 72/295) and C>A conversion (17.97%, 53/295) (**Supplementary Figure 1A**). A consistent trend was also shown in the ctDNA mutation annotation (**Supplementary Figure 1B**). Fisher’s test identified moderate co-occurrence of *TP53* mutations and *TSC2* mutations in both tumor tissues and ctDNA analysis (*p* < 0.1) (**Figures 2E, F**), which was consistent with previous literature (16). Additionally, mutant genes underwent oncogenic pathway enrichment in both tumor tissues and ctDNA samples and demonstrated analogous results such as the NRF2 pathway and the TP53 pathway (**Supplementary Figure 3**) .

### Association Between Genomic Alterations of Tumor Tissues and MVI or Recurrence Status

**Figure 3:**
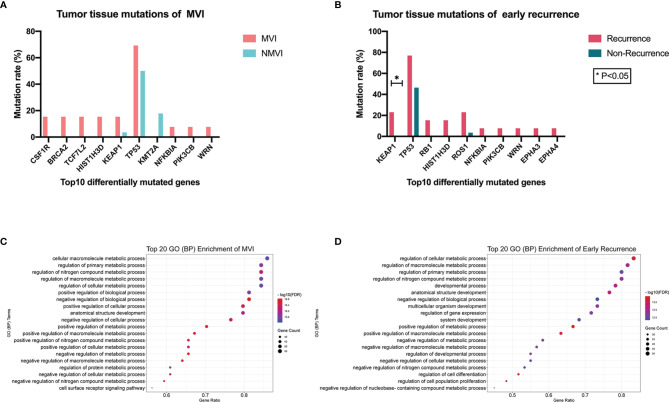
**(A)** Top 10 differentially mutated genes sorted according to *p*-value between MVI and NMVI groups reflecting association between genomic alterations of tumor tissues and MVI status. **(B)** Top 10 differentially mutated genes sorted according to *p*-value between recurrence and non-recurrence groups reflecting association between genomic alterations of tumor tissues and early-recurrence status. **(C, D)** illustrated the functional process enrichment of top 20 GO (BP) enriched terms in MVI patients and early-recurrence patients, respectively.

We compared the mutant genes between MVI and NMVI groups in tissue specimens and obtained the top ten differentially mutated genes. As illustrated, the majority of genes had higher mutation incidence in the MVI group than its counterpart (**Figure 3A**). Meanwhile, mutated genes were compared between early-recurrence and non-recurrence groups. The mutant incidence differences of genes like *KEAP1, TP53*, *HIST1H3D*, *NFKBIA*, *PIK3CB*, and *WRN* reached the consistent tendency between groups both related to MVI and early recurrence. Specifically, the mutant frequency of *KEAP1* in the recurrence group was notably higher than that in the non-recurrence group (23.08% vs. 0%, *p* = 0.027) (**Figure 3B**).

Mutated genes in tumor tissues of MVI patients and early-recurrence patients underwent GO (biological process, BP) and KEGG pathway enrichment, respectively. Significantly enriched GO terms and KEGG pathways with FDR <0.05 were screened out and the top 20 GO (BP) enriched terms of MVI and that of early recurrence patients are demonstrated in **Figures 3C, D** respectively. We observed that common GO terms in both MVI and early recurrence groups were focused on metabolic process.

**Figure 4:**
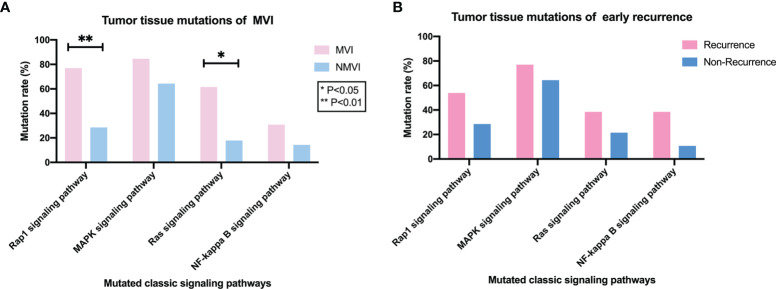
Association between classic signaling pathways alterations in tumor tissues and MVI or early-recurrence status. **(A)** Comparison of mutation frequency of classic tumor pathways (i.e., Rap1, MAPK, Ras, and NF-kappa B signaling pathways) between MVI and NMVI patients. **(B)** Comparison of mutation frequency of Rap1, MAPK, Ras and NF-kappa B signaling pathways between early-recurrence and non-recurrence patients.

Analysis of mutation frequency in KEGG classic tumor pathways (i.e., Rap1, Ras, MAPK, and NF-kappa B signaling pathways) correlated to tumor invasion and metastasis was further conducted, by comparing the alteration rate differences of mapped mutated genes to each pathway. We found that alteration rates of Rap1 (mapped genes *FLT4, KDR, IGF1R, CSF1R, KIT, PIK3CB, FGF19, CTNNB1*, and *GRIN2A*) and Ras (mapped genes *FLT4, KDR, IGF1R, CSF1R, KIT, PIK3CB, FGF19, NF1*, and *GRIN2A*) signaling pathways were significantly higher in the MVI group than the NMVI one with 10/13 (76.92%) vs. 8/28 (28.57%) (*p* = 0.004) and 8/13 (61.54%) vs. 5/28 (17.86%) (*p* = 0.015), respectively (**Figure 4A**). Meanwhile, the similar difference tendency was observed between early recurrence patients and non-recurrence patients, with 7/13 (53.85%) vs. 8/28 (28.57%) in Rap1 pathway alterations and 5/13 (38.46%) vs. 6/28 (21.43%) in Ras pathway alterations, respectively (**Figure 4B**). Furthermore, the correlation between alteration of Rap1 or Ras pathway mapped genes and DFS can be validated in the cBioPortal large cohort (**Supplementary Figure 4**).

### Correlation Between Genomic Alterations of ctDNA and MVI or Recurrence Status

**Figure 5:**
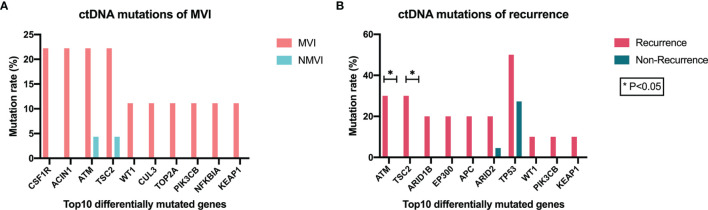
Association between genomic alterations of ctDNA and MVI or recurrence status. **(A)** Top 10 differentially mutated genes sorted according to *p*-value between MVI and NMVI groups. **(B)** Top 10 differentially mutated genes sorted according to *p*-value between recurrence and non-recurrence groups.

Mutant genes in plasma samples were also compared in both MVI/NMVI groups and recurrence/non-recurrence groups, and the top ten differentially mutated genes are shown in **Figure 5**. Among them, *ATM, TSC2, WT1, PIK3CB*, and *KEAP1* reached consistent difference tendency in MVI/NMVI or early-recurrence/non-recurrence comparison, especially *ATM* and *TSC2* (*p* = 0.024 in early-recurrence/non-recurrence groups). *KEAP1* and *PIK3CB* alterations were also in accordance with the difference tendency in tumor tissue samples.

**Figure 6:**
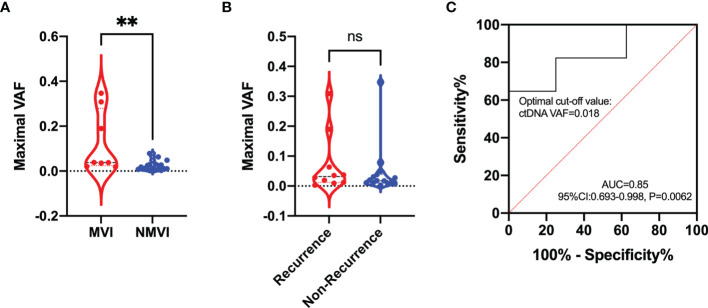
Comparison of the maximal VAF of ctDNA between **(A)** MVI and NMVI groups; **(B)** recurrence and non-recurrence groups. **(C)** ROC curve indicating the performance of ctDNA maximal VAF in determining the presence of MVI in plasma samples. ** indicates p-value < 0.01; ns indicates p-value > 0.05.

The maximal VAF of ctDNA exhibited superior level in MVI patients compared with NMVI patients (0.038 vs. 0.012, *p* = 0.0048) (**Figure 6A**). Analogous difference was also found in early-recurrence and non-recurrence patients though no statistical significance existed (0.032 vs. 0.018, *p* = 0.4284) (**Figure 6B**). To evaluate the performance of ctDNA maximal VAF in preoperative prediction of MVI, the ROC analysis was conducted. The AUC was 0.85 (95% CI 0.693–0.998, *p* = 0.0062) and the cutoff value of ctDNA maximal VAF was set as 0.018 with a sensitivity of 64.71% and a specificity of 100% (**Figure 6C**). Therefore, when ctDNA maximal VAF >0.018 in patients, they were defined as high-risk MVI.

## Discussion

Herein, our research investigated the potential function of genomic mutations and gene mapped pathway alterations to microvascular invasion and early recurrence of HCC patients. The status of MVI is closely related to early recurrence of patients after resection. When it came to alterations of several genes such as *KEAP1* and *PIK3CB*, the tendency of discrepancy was unanimous in MVI/NMVI to recurrence/non-recurrence comparison, not only in tumor tissue but also in ctDNA analysis. Remarkably, alterations in several enriched pathways, associated with angiogenesis, invasion, and metastasis, indicated potential function to MVI and early recurrence. Besides, elevated ctDNA maximal VAF could possibly predict the presence of MVI with good performance, which further enhanced the potential of genomic mutations as promising biomarkers for invasion and metastasis.

The presence of MVI can predict the risk of tumor recurrence and survival after surgery (20, 21). As reported by Yamashita et al., the 1-year recurrence rates were 7.5% and 23.3% for patients without or with MVI, respectively (*p* = 0.01) (22). Furthermore, surgical resection decision with a wide margin could better eradicate MVI adjacent to the tumor, indicating the dramatical impact of predicting the presence of MVI before hepatectomy. As solid malignancies, HCC has a poor rate of excision and a high recurrence rate (23). The urgent conditions prompt us to develop and improve early screening methods applied for patients who are likely to relapse, reminding clinicians to adjust remedies in time. Song et al. have found that *TSC2* mutations were significantly associated with MVI in HCC patients and were independently associated with recurrence within 1 year and poorer RFS after hepatectomy (16). In this study, we focused on more genomic mutations extensively, and found mutations of several genes and mapped signaling pathways reached consistent difference tendency not only in MVI patients but also in 1-year recurrence ones. These results suggested promising value of genomic mutations for predicting status of invasion and metastasis in early-stage HCC patients.

In tumor tissue analysis, *KEAP1, HIST1H3D, TP53, NFKBIA, PIK3CB*, and *WRN* gene mutations had consistent disparity tendency in MVI/NMVI and early recurrence/non-recurrence groups. *KEAP1* and *TP53* genes have co-occurrence tendency in HCC patients in the cBioPortal cohort, and mutation of *TP53* is closely correlated with poorer HCC DFS (log-rank test *p*-value =1.365e-3) (**Supplementary Figure 2B**). As reported by Cleary et al., the presence of *KEAP1* mutations is associated with decreased DFS of HCC patients though lacks sufficient statistical power (24), which is consistent with our results. However, *HIST1H3D, NFKBIA, PIK3CB*, and *WRN* gene mutations have not been reported to be associated with HCC recurrence before, deserving further mechanism analysis.

Though some gene mutations of MVI and early recurrence patients are not exactly the same, they can map to similar functional pathways when performed pathway enrichment. A tumor sample is considered to alter in a given pathway if one or more genes in the pathway contain a recurrent or known driver alteration (25). Finally, alterations of Rap1 and Ras signaling pathways had higher frequency in MVI as well as early-recurrence patients than counterparts. Ras-associated protein-1 (Rap1) is required for angiogenesis during development, which is essential for tubular structure formation (26). Through its interaction with other proteins, Rap1 signaling plays many roles during cell invasion and metastasis in different cancers by regulating cell adhesion, and modulates expression of matrix metalloproteinases or cell proliferation (27). Ras signaling is tightly controlled through a series of post-transcriptional mechanisms and establishes key metabolic and immunologic states supporting cancer migration and metastasis. Overall, these signaling pathways are closely related to tumor invasion and metastasis; thus, gene alteration clusters on these pathways are likely to become novel biomarkers predicting MVI and early recurrence. *PIK3CB*, which shows consistent difference tendency in MVI/NMVI and early recurrence/non-recurrence patient comparison, is in Rap1 and Ras signaling pathways. Previously, researchers have revealed that mutation of *PIK3CB* increased cancer cell proliferation and promote tumorigenic growth (28, 29). Such gene mutations and signaling pathway alterations have great potential to predict early prognosis for HCC patients after hepatectomy.

Recently, studies utilizing ctDNA detection to predict MVI with satisfactory efficacy provide inspiration to us to explore MVI prediction biomarkers. According to Wang et al., ctDNA AF was the independently potential biomarker predicting the presence of MVI before surgery in HCC patients and was associated with recurrence‐free survival (30). Similarly, Wang et al. found that patients with increased mutant allele frequency (MAF) had more incidences of MVI and recurrence (31). Our research results verified the above conclusions that the maximal VAF of ctDNA could expediently distinguish whether there was the presence of MVI in HCC patients before hepatectomy. These results indicated that when MVI occurred in HCC patients, ctDNA was easier to enter into peripheral blood. Besides, VAF suggested the frequency of cancer clones harboring the specific variant in the primary lesion and metastasis, which could be a surrogate for tumor burden (32). What is more, ctDNA genomic alterations such as *KEAP1* and *PIK3CB* were also in accordance with the difference tendency in tumor tissue samples, indicating that the plasma ctDNA can potentially reflect tumor-derived genomic mutation.

However, it is worth noting that still parts of plasma or tissue mutations are private to specific samples, which may be due to tumor heterogeneity and subclonal evolution. An undetectable amount of ctDNA may present in early-stage tumor due to the low release capacity, which leads to missed detection of tumor mutations in ctDNA. Therefore, the improvement of multi-region sampling and standardized sequencing platforms is necessary to fully reflect HCC genomic landscape. What is more, the primary limitation of our research is the moderate sample size, the conclusions need to be validated in a larger cohort size, and the mechanisms of the mutations and the pathways on MVI need to be explored in the future. Though these limitations exist, we provide evidence that somatic alterations in early-stage HCC patients can be detected with low abundance and can be relatively promising biomarkers to predict early invasion and metastasis. In clinical practice, we hope that genomic mutations can guide the choice of selecting appropriate type of surgery or adjuvant treatment of HCC patients, and enable early detection of postoperative recurrence to help surveillance and prognostic management.

## Conclusion

Genomic mutations have potential to predict preoperative MVI and postoperative early recurrence in early-stage HCC patients, which include tumor tissue and ctDNA alterations. In addition, alterations of enriched signaling pathways can also serve as indicators of prediction. The integration of differentially significant mutated genes and ctDNA VAF in the surveillance and management of operable HCC may achieve better outcomes for patients.

## Data Availability Statement

The data analyzed in this study is subject to the following restrictions: It should be in accordance with the Regulations on the Management of Human Genetic Resources of China and the provisions of the Scope and Procedures for the Recordation of the Provision or Open Use of Human Genetic Resources in China. Requests to access these datasets should be directed to BY/West China Hospital of Sichuan University, yingbinwu@scu.edu.cn.

## Ethics Statement

The studies involving human participants were reviewed and approved by the Biomedical Ethics Committee of West China Hospital of Sichuan University [Reference No. 2019 (203)]. The patients/participants provided their written informed consent to participate in this study. Written informed consent was obtained from the individual(s) for the publication of any potentially identifiable images or data included in this article.

## Author Contributions

ZX Investigation, Conceptualization, Methodology, Formal analysis, Writing—original draft, Writing—review & editing, and Visualization. JL Conceptualization, Methodology, Formal analysis, Validation, Writing—original draft, and Writing—review & editing. ZX and JL contributed equally to this work. HZ Investigation, Conceptualization, and Resources. YZ Methodology and Writing—review & editing. JS Investigation and Methodology. PC Methodology and Validation. LB Methodology and Formal analysis. HC Investigation and Methodology. JZ Conceptualization, Resources, Data Curation, Supervision, and Writing—review & editing. JC Supervision, Writing—review & editing, and Funding acquisition. BY Conceptualization, Resources, Data Curation, Supervision, Project administration, Writing—review & editing, and Funding acquisition. All authors contributed to the article and approved the submitted version.

## Funding

This work was funded by the National Natural Science Foundation of China [81873979], the Project of Science and Technology Department of Sichuan Province [2020YJ0106], and the Project of Science and Technology Department of Sichuan Province [2020YJ0049].

## Conflict of Interest

The authors declare that the research was conducted in the absence of any commercial or financial relationships that could be construed as a potential conflict of interest.

## Publisher’s Note

All claims expressed in this article are solely those of the authors and do not necessarily represent those of their affiliated organizations, or those of the publisher, the editors and the reviewers. Any product that may be evaluated in this article, or claim that may be made by its manufacturer, is not guaranteed or endorsed by the publisher.

## Abbreviations

MVI Microvascular invasion; ctDNA Circulating tumor DNA; VAF Variant allele frequency; AFP Alpha fetoprotein; RFS Recurrence free survival; GO Gene Ontology; KEGG Kyoto Encyclopedia of Genes and Genomes; FDR False discovery rate; ROC Receiver operating characteristic curve; BCLC Barcelona Clinic Liver Cancer; TCGA The Cancer Genome Atlas; AST Aspartate aminotransferase; ALT Alanine aminotransferase; PIVKA-II Protein Induced by Vitamin K Absence or Antagonist-II; CEA Carcinoembryonic antigen; PBL Peripheral blood leukocyte.
