# Supplementary material for: Cancer Genomic Alterations Can Be Potential Biomarkers Predicting Microvascular Invasion and Early Recurrence of Hepatocellular Carcinoma
Source: Front Oncol. 2022 Jan 27;12:783109. doi: 10.3389/fonc.2022.783109 (PMC8828586; doi:10.3389/fonc.2022.783109)
Supplement: Supplementary file 1 [file DataSheet_1.pdf]

Supplementary Material

1 Supplementary Figures and Tables

1.1 Supplementary Figures

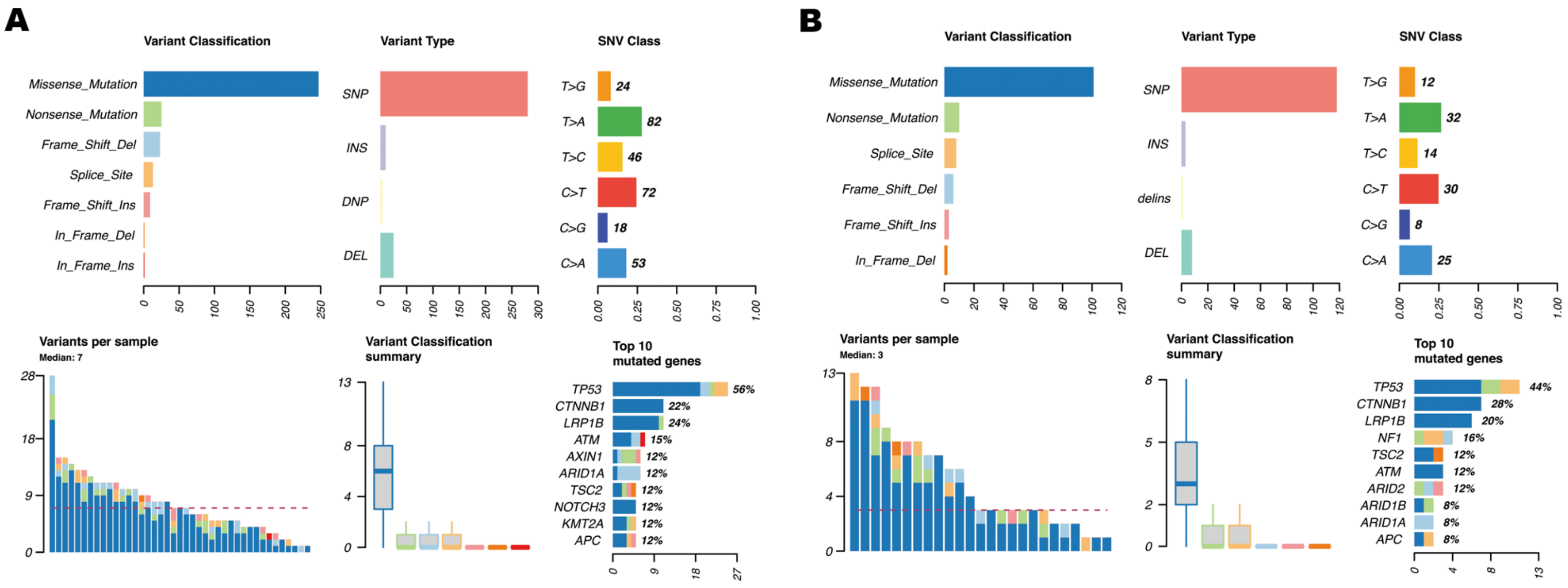

Supplementary Figure 1. (A) and (B) are MAF summary plots of tumor tissue samples and ctDNA samples in our HCC patients, respectively. The combination plots include variant classification, variant type, SNV class with different colors and variants per sample as well as top 10 mutated genes in tumor tissues and ctDNA, respectively.

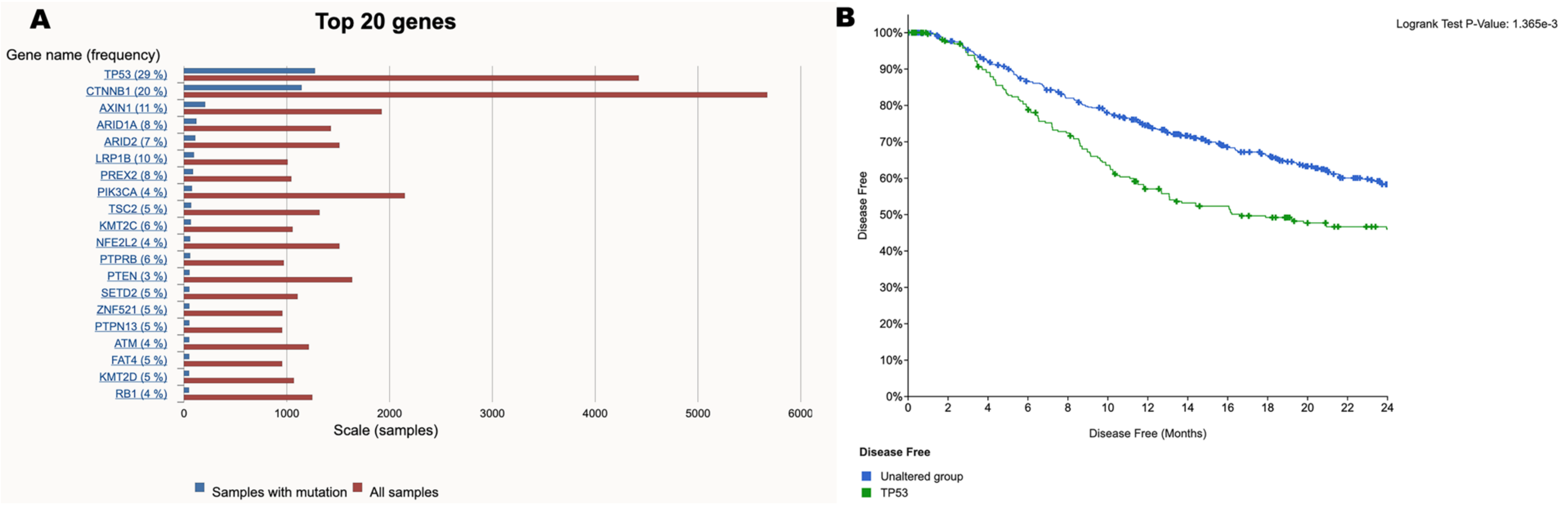

Supplementary Figure 2. (A) Top 20 mutant genes of hepatocellular carcinoma queried by tissue type and histology from the COSMIC database (as of July 24, 2021). **(B)** Mutation of *TP53* gene is closely correlated with poorer DFS (log-rank test P value =1.365e-3) of hepatocellular carcinoma patients in cBioPortal database (Querying 1468 patients / 1488 samples in 7 studies).

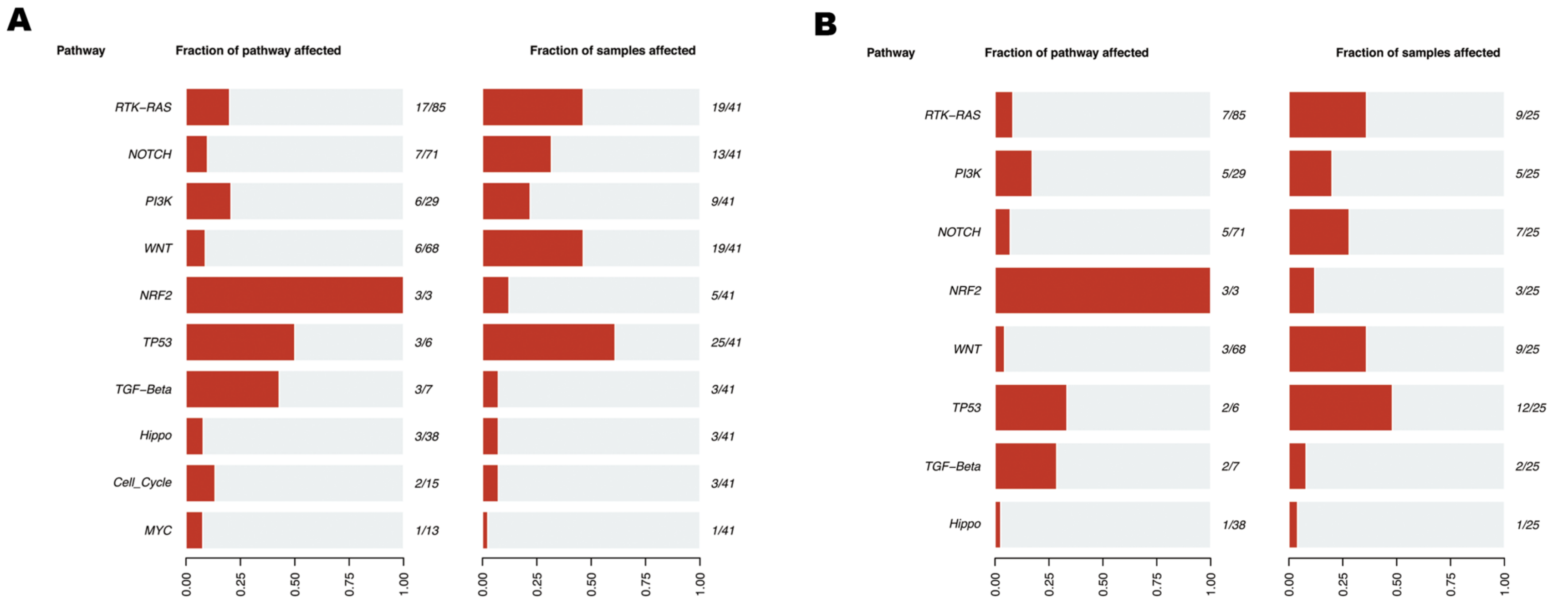

Supplementary Figure 3. (A) and **(B)** illustrate enriched oncogenic pathways of mutant genes in tumor tissue samples and ctDNA samples, respectively.

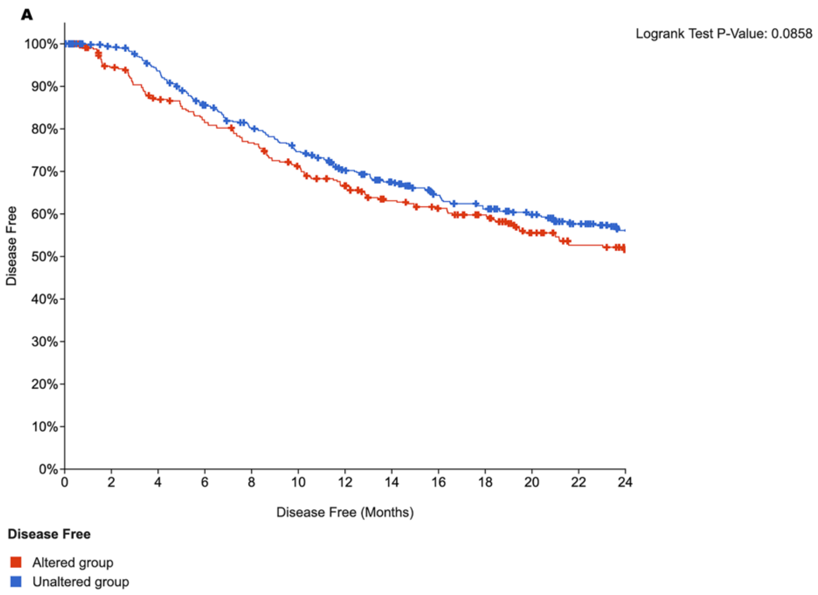

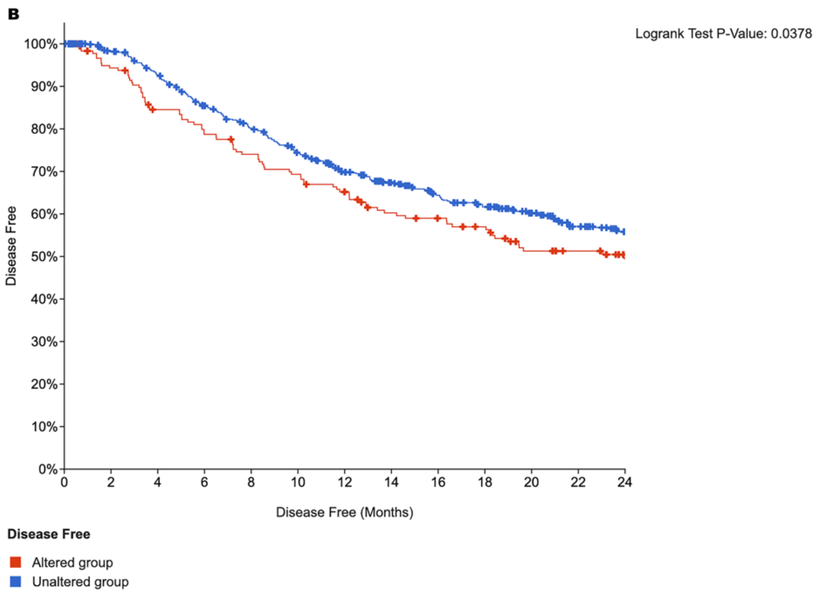

Supplementary Figure 4. (A) Mutation of Rap1 signaling pathway mapped genes (*FLT4, KDR, IGF1R, CSF1R, KIT, PIK3CB, FGF19, CTNNB1, GRIN2A*) is closely correlated with poorer DFS (log-rank test P value =0.0858) of hepatocellular carcinoma patients in cBioPortal database (Querying 1468 patients / 1488 samples in 7 studies). **(B)** Mutation of Ras signaling pathway mapped genes (*FLT4, KDR, IGF1R, CSF1R, KIT, PIK3CB, FGF19, NF1, GRIN2A*) is closely correlated with poorer DFS (log-rank test P value =0.0378) of hepatocellular carcinoma patients in cBioPortal database (Querying 1468 patients / 1488 samples in 7 studies).

1.2 Supplementary Tables

Supplementary Table 1. Detailed information of sequencing samples

Sample ID	Tumor tissue mutations	ctDNA mutations	Tumor diameter (cm)	Differentiation	Liver cirrhosis	MVI	MVI number	Early recurrence (≤12 months)	Recurrence area	RFS (months)	Postoperative adjuvant therapy
1	Yes	Yes	4.7	Moderate	No	No	-	No		23.27	No
2	Yes	Yes	3.0	Moderate	No	No	-	No		23.23	No
4	Yes	Yes	7.0	Moderate	No	No	-	No		15.70	No
5	Yes	Yes	6.0	Moderate	No	Yes	≤5	No		22.73	TACE after recurrence
6	Yes	Yes	2.8	Moderate	No	No	-	No		22.53	No
7	Yes	Yes	6.2	Moderate	No	Yes	≤5	No		22.07	Hepatic resection after recurrence
9	Yes	Yes	3.5	Moderate	No	No	-	No		22.47	No
12	Yes	Yes	3.5	Moderate	Yes	No	-	No		21.77	No
15	Yes	Yes	2.2	Moderate	Yes	No	-	No		21.10	No
16	Yes	Yes	8.5	Moderate	Yes	Yes	≤5	Yes	Intrahepatic	7.40	No
18	Yes	Yes	2.4	Poor	No	No	-	No		18.37	No
19	Yes	Yes	1.3	Moderate	Yes	Yes	≤5	Yes	Intrahepatic	12.03	No
20	**NA***	Yes	4.9	Moderate	No	Yes	＞5	Yes	Intrahepatic	4.00	TACE after recurrence

Sample ID	Tumor tissue mutations	ctDNA mutations	Tumor diameter (cm)	Differentiation	Liver cirrhosis	MVI	MVI number	Early recurrence (≤12 months)	Recurrence area	RFS (months)	Postoperative adjuvant therapy
21	Yes	Yes	8.0	Moderate	Yes	Yes	≤5	No		20.83	TACE
22	**NA***	**NA***	1.8	Poor	Yes	No	-	No		16.07	No
24	Yes	Yes	8.0	Poor	No	No	-	No		20.67	No
28	Yes	Yes	4.3	Poor	No	No	-	Yes	Intrahepatic	1.37	Radiofrequency ablation after recurrence
29	Yes	Yes	2.0	Moderate	No	No	-	No		20.47	No
30	Yes	Yes	3.5	Moderate	Yes	No	-	Yes	Intrahepatic	10.20	Radiofrequency ablation after recurrence
31	Yes	Yes	2.7	Moderate	No	No	-	No		20.43	No
32	Yes	Yes	8.0	Moderate	No	No	-	Yes	Extrahepatic	8.60	Hepatic resection after recurrence
33	Yes	Yes	6.0	Moderate	No	No	-	No		20.37	No
34	Yes	Yes	2.8	Poor	No	No	-	Yes	Intrahepatic	8.67	TACE after recurrence
35	Yes	Yes	16.2	Moderate	Yes	Yes	≤5	No		20.07	TACE
36	Yes	Yes	2.7	Poor	No	No	-	No		19.73	No
38	Yes	Yes	8.0	Moderate	Yes	No	-	**NA**	**NA**	**NA**	No
39	Yes	Yes	2.5	Moderate	Yes	No	-	No		19.70	No

Sample ID	Tumor tissue mutations	ctDNA mutations	Tumor diameter (cm)	Differentiation	Liver cirrhosis	MVI	MVI number	Early recurrence (≤12 months)	Recurrence area	RFS (months)	Postoperative adjuvant therapy
40	Yes	Yes	5.9	Moderate	Yes	No	-	No		19.50	No
41	Yes	Yes	1.9	Poor	Yes	No	-	No		19.27	No
43	Yes	**NA***	2.5	Moderate	Yes	No	-	No		19.27	No
45	Yes	**NA***	2.5	Moderate	No	No	-	No		18.80	NA
46	Yes	Yes	12.6	Poor	No	Yes	＞5	Yes	Extrahepatic	3.13	NA
47	Yes	Yes	3.0	Poor	No	No	-	No		18.70	No
52	Yes	Yes	4.0	Moderate	Yes	No	-	No		18.03	No
54	Yes	Yes	6.5	Poor	Yes	No	-	Yes	Intrahepatic	11.53	Radiofrequency ablation after recurrence
55	Yes	**NA***	6.5	Moderate	No	No	-	No		17.87	No
56	Yes	Yes	16.0	Poor	No	Yes	＞5	Yes	Intrahepatic	8.90	Sorafenib; TACE after recurrence
57	Yes	**NA***	5.5	Poor	No	Yes	＞5	Yes	Intrahepatic	3.43	Sorafenib; TACE
58	Yes	**NA***	8.0	Poor	Yes	Yes	≤5	Yes	Extrahepatic	7.83	No
59	Yes	**NA***	4.0	Moderate	No	No	-	No		17.60	No
60	Yes	**NA***	5.5	Moderate	Yes	No	-	No		17.53	No
61	Yes	**NA***	7.5	Poor	No	Yes	≤5	No		17.43	No

Sample ID	Tumor tissue mutations	ctDNA mutations	Tumor diameter (cm)	Differentiation	Liver cirrhosis	MVI	MVI number	Early recurrence (≤12 months)	Recurrence area	RFS (months)	Postoperative adjuvant therapy
86	Yes	**NA***	5.8	Moderate	No	Yes	-	Yes	Intrahepatic	4.97	Radiofrequency ablation and TACE after recurrence
88	Yes	**NA***	15.0	Poor	No	Yes	-	Yes	Intrahepatic	2.77	TACE after recurrence

NA* indicates that the sequencing process was not performed or failed due to insufficient sample size

NA indicates that the No.38 patient was lost to follow-up

Supplementary Table 2. List of human tumor-associated 1021-gene panel

**All 4847 exon regions of 312 genes**									
ABL1	*ACVR1B*	*AKT1*	*AKT2*	*AKT3*	*ALK*	*APC*	*AR*	*ARAF*	*ARID1A*
ARID1B	*ARID2*	*ASXL1*	*ATM*	*ATR*	*ATRX*	*AURKA*	*AURKB*	*AXIN1*	*AXIN2*
AXL	*B2M*	*BAP1*	*BARD1*	*BCL2*	*BCL2L1*	*BCOR*	*BLM*	*BMPR1A*	*BRAF*
BRCA1	*BRCA2*	*BRD4*	*BRIP1*	*BTK*	*CARD11*	*CASP8*	*CBFB*	*CBL*	*CCND1*
CCND2	*CCND3*	*CCNE1*	*CD274*	*CDC73*	*CDH1*	*CDK12*	*CDK4*	*CDK6*	*CDK8*
CDKN1A	*CDKN1B*	*CDKN2A*	*CDKN2B*	*CDKN2C*	*CEBPA*	*CHEK1*	*CHEK2*	*CIC*	*CREBBP*
CRKL	*CSF1R*	*CTCF*	*CTNNA1*	*CTNNB1*	*CUL3*	*CYLD*	*DAXX*	*DDR1*	*DDR2*
DICER1	*DNMT3A*	*DOT1L*	*EGFR*	*EIF1AX*	*C11orf30*	*EP300*	*EPAS1*	*EPCAM*	*EPHA2*

EPHA3	*EPHA5*	*EPHB1*	*EPHB6*	*ERBB2*	*ERBB3*	*ERBB4*	*ERCC1*	*ERCC3*	*ERCC4*
ERCC5	*ERG*	*ERRFI1*	*ESR1*	*EXT1*	*EXT2*	*EZH2*	*FAM123B*	*FAM175A*	*FANCA*
FANCC	*FANCD2*	*FANCE*	*FANCF*	*FANCG*	*FANCL*	*FANCM*	*FAS*	*FAT1*	*FAT2*
FBXW7	*FGF19*	*FGF3*	*FGF4*	*FGFR1*	*FGFR2*	*FGFR3*	*FGFR4*	*FH*	*FLCN*
FLT1	*FLT3*	*FLT4*	*FOXA1*	*FOXL2*	*FOXP1*	*FUBP1*	*GALNT12*	*GATA3*	*GNA11*
GNAQ	*GNAS*	*GRIN2A*	*GRM3*	*HDAC1*	*HGF*	*HNF1A*	*HOXB13*	*HRAS*	*IDH1*
IDH2	*IFNG*	*IFNGR1*	*IGF1R*	*IKBKE*	*IKZF1*	*IL7R*	*INPP4B*	*IRF2*	*IRS2*
JAK1	*JAK2*	*JAK3*	*JUN*	*KDM5A*	*KDM5C*	*KDM6A*	*KDR*	*KEAP1*	*KIT*
KRAS	*LRP1B*	*MAF*	*MAP2K1*	*MAP2K2*	*MAP2K4*	*MAP3K1*	*MAPK1*	*MAX*	*MCL1*
MDM2	*MDM4*	*MED12*	*MEF2B*	*MEN1*	*MET*	*MITF*	*MLH1*	*MLH3*	*MLL*
MLL2	*MLL3*	*MPL*	*MRE11A*	*MS4A1*	*MSH2*	*MSH3*	*MSH6*	*MST1R*	*MTOR*
MUTYH	*MYC*	*MYCL1*	*MYCN*	*MYD88*	*NBN*	*NCOR1*	*NF1*	*NF2*	*NFE2L2*
NFKBIA	*NKX2-1*	*NOTCH1*	*NOTCH2*	*NOTCH3*	*NPM1*	*NRAS*	*NSD1*	*NTHL1*	*NTRK1*
NTRK2	*NTRK3*	*PALB2*	*PARK2*	*PARP1*	*PAX5*	*PBRM1*	*PCK1*	*PDCD1*	*PDCD1LG2*
PDGFRA	*PDGFRB*	*PDK1*	*PIK3CA*	*PIK3CB*	*PIK3CG*	*PIK3R1*	*PIK3R2*	*PMS1*	*PMS2*
POLD1	*POLE*	*POT1*	*PPP2R1A*	*PRDM1*	*PRKAR1A*	*PTCH1*	*PTCH2*	*PTEN*	*PTPN11*
PTPRD	*RAC1*	*RAD50*	*RAD51*	*RAD51B*	*RAD51C*	*RAD51D*	*RAD52*	*RAD54L*	*RAF1*

RARA	*RB1*	*RBM10*	*RECQL*	*RECQL4*	*RET*	*RHOA*	*RICTOR*	*RINT1*	*RNF43*
ROS1	*RPTOR*	*RUNX1*	*SDHA*	*SDHAF2*	*SDHB*	*SDHC*	*SDHD*	*SERPINB3*	*SERPINB4*
SETD2	*SF3B1*	*SLX4*	*SMAD2*	*SMAD3*	*SMAD4*	*SMARCA4*	*SMARCB1*	*SMO*	*SOCS1*
SOX2	*SOX9*	*SPOP*	*SRC*	*STAG2*	*STAT3*	*STK11*	*SUFU*	*SYK*	*TBX3*
TCF7L2	*TERC*	*TET2*	*TGFBR2*	*TMEM127*	*TMPRSS2*	*TNFAIP3*	*TNFRSF14*	*TOP1*	*TOP2A*
TP53	*TSC1*	*TSC2*	*TSHR*	*U2AF1*	*VEGFA*	*VHL*	*WRN*	*WT1*	*XPO1*
XRCC2	*ZMAT3*								

Introns, promoters, and fusion breakpoint regions of 38 genes

ALK	*BCL2L11*	*BRAF*	*BRCA1*	*BRD4*	*CD74*	*EGFR*	*EML4*	*ERG*	*ETV6*
EZR	*FGFR1*	*FGFR2*	*FGFR3*	*KIF5B*	*KIT*	*MAML2*	*MET*	*MSH2*	*MYC*
MYCL1	*NCOA4*	*NOTCH2*	*NTRK1*	*NTRK2*	*NTRK3*	*PDGFRA*	*RAF1*	*RET*	*ROS1*
RSPO2	*SDC4*	*SLC34A2*	*TERT*	*TFE3*	*TMPRSS2*	*TPM3*	*PMS2*		

1778 coding regions of 709 other related genes

ABCA13	*ABCB1*	*ABCC1*	*ABCC11*	*ABCC2*	*ABCG2*	*ABL2*	*ACACA*	*ACIN1*	*ACTB*
ACTG1	*ACTG2*	*ACVR2A*	*ACVRL1*	*ADAM29*	*ADAMTS5*	*ADCY1*	*AFF1*	*AFF2*	*AFF3*
AHNAK	*AKAP9*	*ALB*	*AMOT*	*ANGPT1*	*ANK3*	*ANKRD11*	*ANKRD30A*	*ANKRD30B*	*APEX1*
APOBEC3B	*ARAP3*	*ARFGEF1*	*ARFGEF2*	*ARHGAP29*	*ARHGAP35*	*ARID4B*	*ARID5B*	*ARNT*	*ASCL4*

ASH1L	*ASMTL*	*ASPM*	*ASTN1*	*ASXL2*	*ATIC*	*ATP11B*	*ATP12A*	*ATP1A1*	*ATP2B3*
BAZ2B	*BBC3*	*BBS9*	*BCAS1*	*BCL10*	*BCL11A*	*BCL11B*	*BCL2A1*	*BCL2L11*	*BCL3*
BCL6	*BCL9*	*BCORL1*	*BCR*	*BIRC3*	*BMPR2*	*BNC2*	*BPTF*	*BRD2*	*BRD3*
BRSK1	*BRWD1*	*BTLA*	*BUB1*	*C15orf23*	*C15orf55*	*C1QA*	*C1S*	*C3orf70*	*C7orf53*
C8orf34	*CACNA1E*	*CADM2*	*CALR*	*CAMTA1*	*CASP1*	*CASQ2*	*CBLB*	*CBR1*	*CBR3*
CCDC168	*CCNA1*	*CCNB3*	*CCT3*	*CCT5*	*CCT6B*	*CD22*	*CD33*	*CD5L*	*CD74*
CDA	*CDH11*	*CDH18*	*CDH23*	*CDK13*	*CHD1*	*CHD1L*	*CHD4*	*CHD6*	*CHD8*
CHD9	*CHFR*	*CHI3L1*	*CHN1*	*CIITA*	*CLDN18*	*CLP1*	*CLSPN*	*CLTC*	*CNOT3*
CNOT4	*CNTN1*	*CNTN5*	*CNTNAP1*	*CNTNAP5*	*COL1A1*	*COL2A1*	*COL5A1*	*COL5A2*	*COL5A3*
COPS2	*CPS1*	*CRIPAK*	*CRLF2*	*CRNKL1*	*CRTC1*	*CSF1*	*CSF3R*	*CSMD1*	*CSMD3*
CSNK1A1	*CSNK1G3*	*CTLA4*	*CTNNA2*	*CTNND1*	*CUX1*	*CXCR4*	*CYBA*	*CYP19A1*	*CYP1A1*
CYP1B1	*CYP2A13*	*CYP2C8*	*CYP2D6*	*CYP3A4*	*CYP3A5*	*DCC*	*DDX3X*	*DDX5*	*DEK*
DHX35	*DHX9*	*DIAPH1*	*DIS3L2*	*DLC1*	*DMD*	*DNAH6*	*DNAJB1*	*DNM2*	*DNMT1*
DNMT3B	*DOCK2*	*DOCK7*	*DPYD*	*DRGX*	*DTX1*	*DUSP22*	*DYSF*	*E2F3*	*EBF1*
ECT2L	*EED*	*EEF1A1*	*EGFL7*	*EGR3*	*EIF2AK3*	*EIF2C3*	*EIF3A*	*EIF4A2*	*EIF4G3*
ELAC2	*ELF1*	*ELF3*	*ELMO1*	*ELN*	*EME2*	*EMID2*	*EML4*	*EPC1*	*EPHA1*
EPHA4	*EPHA7*	*EPHB2*	*EPHB4*	*EPOR*	*EPPK1*	*EPS15*	*ERBB2IP*	*ERCC2*	*ESR2*

ETS1	*ETV1*	*ETV5*	*ETV6*	*EWSR1*	*EZR*	*F8*	*FAM131B*	*FAM135B*	*FAM157B*
FAM46C	*FAM5C*	*FAP*	*FASLG*	*FAT3*	*FAT4*	*FCGR1A*	*FCGR2A*	*FCGR2B*	*FCGR3A*
FCRL4	*FGF10*	*FGF12*	*FGF14*	*FGF23*	*FGF6*	*FLG*	*FLI1*	*FLNC*	*FMN2*
FN1	*FNDC4*	*FOXA2*	*FOXO1*	*FOXO3*	*FOXQ1*	*FRMPD4*	*FUS*	*FXR1*	*FYN*
FZD1	*G3BP1*	*G3BP2*	*GAB2*	*GABRA6*	*GATA1*	*GATA2*	*GFRAL*	*GIGYF1*	*GKN2*
GLB1L3	*GLI1*	*GLI2*	*GLI3*	*GMPS*	*GNA13*	*GNG2*	*GPC3*	*GPR124*	*GPS2*
GPX1	*GRB7*	*GSK3B*	*GSTM5*	*GSTP1*	*GUSB*	*H3F3A*	*H3F3B*	*H3F3C*	*HCLS1*
HCN1	*HDAC4*	*HDAC9*	*HECW1*	*HEY1*	*HIST1H1C*	*HIST1H1D*	*HIST1H1E*	*HIST1H2AC*	*HIST1H2AG*
HIST1H2AL	*HIST1H2AM*	*HIST1H2BC*	*HIST1H2BD*	*HIST1H2BJ*	*HIST1H2BK*	*HIST1H2BO*	*HIST1H3B*	*HIST1H3C*	*HIST1H3D*
HIST1H3F	*HIST1H3G*	*HIST1H3H*	*HIST1H3I*	*HIST1H4I*	*HIST3H3*	*HLA-A*	*HLA-B*	*HLA-C*	*HLF*
HMCN1	*HNF1B*	*HNRPDL*	*HOXA11*	*HOXA13*	*HOXA3*	*HOXA9*	*HOXC13*	*HOXD11*	*HOXD13*
HSD3B1	*HSP90AA1*	*HSP90AB1*	*HSPA8*	*HSPD1*	*HSPH1*	*ICK*	*ICOSLG*	*ID3*	*IFITM3*
IGF1	*IGF2*	*IGF2R*	*IGLL5*	*IKZF2*	*IKZF3*	*IL10*	*IL1RAPL1*	*IL21R*	*IL6*
IL6ST	*IMPG1*	*ING1*	*INHBA*	*INPP4A*	*INPPL1*	*INSR*	*IRF4*	*IRF6*	*IRS1*
ITGB3	*ITK*	*ITSN1*	*JARID2*	*KALRN*	*KAT6A*	*KAT6B*	*KCNJ5*	*KCNQ2*	*KDM2B*
KEL	*KIF5B*	*KLF4*	*KLHL6*	*KLK1*	*KRTAP5-5*	*L3MBTL1*	*LAMA2*	*LATS1*	*LATS2*
LCP1	*LEF1*	*LGALS8*	*LIFR*	*LPHN2*	*LPP*	*LRP2*	*LRP4*	*LRP5*	*LRP6*

LRRC7	*LRRK2*	*LYN*	*LZTS1*	*MACF1*	*MAD1L1*	*MAGI2*	*MAML2*	*MAML3*	*MAP3K13*
MAPK3	*MCC*	*MCM3*	*MDC1*	*MECOM*	*MEF2C*	*MGA*	*MIB1*	*MIOS*	*MKL1*
MLL4	*MLLT3*	*MMP11*	*MMP2*	*MN1*	*MNDA*	*MNX1*	*MSH4*	*MSN*	*MSR1*
MTHFR	*MTRR*	*MUC5B*	*MYH11*	*MYH14*	*MYH9*	*MYO3A*	*MYOD1*	*NAP1L1*	*NAV3*
NCAM2	*NCF2*	*NCF4*	*NCK1*	*NCOA3*	*NCOA4*	*NCOR2*	*NCSTN*	*NDUFA13*	*NFATC4*
NFE2L3	*NKX3-1*	*NLRC3*	*NOD1*	*NOS3*	*NOTCH4*	*NQO1*	*NR1I2*	*NR2F2*	*NR4A2*
NRG1	*NRP2*	*NRXN1*	*NTM*	*NUMA1*	*NUP107*	*NUP210*	*NUP93*	*NUP98*	*OBSCN*
OGDH	*OMD*	*OPCML*	*OR11G2*	*OR2T4*	*OR4A15*	*OR4C6*	*OR5L2*	*OR6F1*	*P2RY8*
P4HB	*PABPC1*	*PABPC3*	*PAG1*	*PAK1*	*PAK3*	*PASK*	*PAX3*	*PAX7*	*PC*
PCDH18	*PCSK6*	*PCSK7*	*PDCD11*	*PDE4DIP*	*PDGFB*	*PDILT*	*PER1*	*PGR*	*PHF1*
PHF6	*PIK3C2A*	*PIK3C2B*	*PIK3C2G*	*PIK3C3*	*PIM1*	*PKD1L2*	*PKHD1*	*PLAG1*	*PLCB1*
PLCG1	*PLCG2*	*PLK1*	*PLXNA1*	*PLXNB2*	*PNRC1*	*POLQ*	*POM121*	*POM121L12*	*POU2AF1*
PPM1D	*PPP1R17*	*PPP6C*	*PRDM16*	*PREX2*	*PRF1*	*PRKAA1*	*PRKCB*	*PRKCI*	*PRKDC*
PRRX1	*PRX*	*PSG2*	*PSIP1*	*PSMB1*	*PSMB5*	*PTGS1*	*PTGS2*	*PTPN13*	*PTPN2*
PTPRB	*PTPRK*	*PTPRO*	*PTPRS*	*PTPRT*	*PTPRU*	*RAB35*	*RAC2*	*RAD21*	*RAD54B*
RANBP2	*RASA1*	*RASGRP1*	*RBL1*	*REL*	*RELN*	*RFC1*	*RGS3*	*RHEB*	*RHOH*
RHOT1	*RIT1*	*RNASEL*	*ROBO1*	*ROBO2*	*ROBO3*	*ROCK1*	*RPGR*	*RPS6KB1*	*RPS6KB2*

RSPO2	*RSPO3*	*RUNX1T1*	*RUNX2*	*RXRA*	*RYR1*	*RYR2*	*SBDS*	*SCUBE2*	*SDC4*
SEC31A	*SEMA3A*	*SEMA3E*	*SEMA6A*	*SERPINA7*	*SETBP1*	*SETDB1*	*SF1*	*SF3A1*	*SFPQ*
SGCZ	*SGK1*	*SH2B3*	*SH2D1A*	*SH3PXD2A*	*SHH*	*SI*	*SIN3A*	*SLC16A1*	*SLC1A2*
SLC22A16	*SLC22A18*	*SLC22A2*	*SLC22A3*	*SLC34A2*	*SLCO1B3*	*SLIT1*	*SLIT2*	*SMARCD1*	*SMARCE1*
SMC1A	*SMC1B*	*SNCAIP*	*SNTG1*	*SNX29*	*SOD2*	*SOS1*	*SOX10*	*SOX17*	*SPEN*
SPRR3	*SPSB4*	*SPTA1*	*SRD5A2*	*SRGAP1*	*SRGAP3*	*SRSF2*	*SRSF7*	*STAG1*	*STAT1*
SUCLG1	*SUCLG2*	*SULT1A1*	*SUZ12*	*SVEP1*	*SYNCRIP*	*SYNE1*	*TAF1*	*TAF15*	*TAF1L*
TAL1	*TBL1XR1*	*TBX15*	*TBX22*	*TCEB1*	*TCF12*	*TCF3*	*TCF4*	*TCL1A*	*TEC*
TENM3	*TERT*	*TET1*	*TFDP1*	*TFDP2*	*TFE3*	*TGFBR1*	*THBS2*	*TJP1*	*TLE1*
TLL2	*TLR4*	*TLX3*	*TMEM132D*	*TNFSF11*	*TNN*	*TP53BP1*	*TP63*	*TP73*	*TPM3*
TPR	*TRAF2*	*TRAF7*	*TRIM24*	*TRIM58*	*TRIO*	*TRPC5*	*TRRAP*	*TSHZ2*	*TSHZ3*
TTF1	*TUBA3C*	*TUBB3*	*TUSC3*	*TXNIP*	*TYMS*	*TYR*	*UBE2D2*	*UBR5*	*UGT1A1*
UMPS	*UPF3B*	*USH2A*	*USP6*	*USP8*	*VEZF1*	*VIM*	*VTCN1*	*WASF3*	*WDR90*
WDTC1	*WHSC1*	*WHSC1L1*	*WIPF1*	*WNK1*	*WNT5A*	*WSCD2*	*WWOX*	*WWP1*	*WWP2*
XIAP	*XPC*	*XRCC1*	*XRCC3*	*YAP1*	*YY1AP1*	*ZBTB16*	*ZC3H11A*	*ZFHX3*	*ZFP36L1*
ZFP36L2	*ZFPM2*	*ZIC3*	*ZNF217*	*ZNF384*	*ZNF521*	*ZNF638*	*ZNF750*	*ZNF804B*	

Supplementary Table 3. Surgical details of HCC patients

Variables	HCC patients (N=44)
**Type of resection**	
Anatomical tumorectomy: n (%)	17 (38.64%)
Laparoscopic tumorectomy: n (%)	27 (61.36%)
Blood loss (ml): mean ± SD (Minimum-Maximum)	330 ± 386.86 （30-2000）
Blood transfusion: n (%)	4 (9.09%)
**Operative time (hour)**	
>3: n (%)	32 (72.73%)
3: n (%)	7 (15.91%)
2: n (%)	5 (11.36%)
